# A New Lens on Childhood Obesity: Developing and Validating the Obesity-Related Well-Being (ORWB) Scale for Assessing Well-Being in Obese Children and Adolescents

**DOI:** 10.1155/jobe/2711978

**Published:** 2025-10-10

**Authors:** Jihed Faleh, Ismail Dergaa, Halil İbrahim Ceylan, Hatem Ghouili, Noomen Guelmami, Haitham Jahrami, Khaled Trabelsi, Mohamed Ben Aissa, Makrem Zghibi, Raul Ioan Muntean, Achraf Ammar

**Affiliations:** ^1^High Institute of Sport and Physical Education, University of Sfax, Sfax, Tunisia; ^2^Physical Activity, Sport, and Health, UR18JS01, National Observatory of Sport, Tunis, Tunisia; ^3^High Institute of Sport and Physical Education Ksar Said, University of Manouba, Manouba, Tunisia; ^4^High Institute of Sports and Physical Education of Kef, University of Jendouba, Jendouba, Tunisia; ^5^Department of Physical Education of Sports Teaching, Faculty of Sports Sciences, Atatürk University, Erzurum, Türkiye; ^6^Department of Psychiatry, College of Medicine and Health Sciences, Arabian Gulf University, Manama, Bahrain; ^7^Research Laboratory Education, Motricité, Sport et Santé, EM2S, LR19JS01, High Institute of Sport and Physical Education of Sfax, University of Sfax, Sfax, Tunisia; ^8^Department of Movement Sciences and Sports Training, School of Sport Science, The University of Jordan, Amman, Jordan; ^9^Department of Physical Education and Sport, Faculty of Law and Social Sciences, University “1 Decembrie 1918”, Alba Iulia, Romania; ^10^Department of Training and Movement Science, Institute of Sport Science, Johannes Gutenberg-University Mainz, Mainz, Germany; ^11^Research Laboratory, Molecular Bases of Human Pathology, LR19ES13, Faculty of Medicine of Sfax, University of Sfax, Sfax 3000, Tunisia

**Keywords:** behavioral health, childhood obesity, health surveys, intervention evaluation, nutrition, obesity assessment, psychosocial effects, quality measurement

## Abstract

The increasing prevalence of pediatric obesity is a significant public health issue, with conventional diagnostic methods often overlooking its psychological, social, and lifestyle consequences. This study aimed to create and confirm the validity of the obesity-related well-being (ORWB) scale, a new tool designed to evaluate the diverse impacts of obesity on children and adolescent's physical, psychological, social, and lifestyle well-being. The scale was developed through extensive literature analysis and consultations with experts in the field. The study included 672 students aged 12–18 years, predominantly overweight, from a pool of 19,876 students across four Tunisian governorates. The scale's internal consistency was evaluated using robust measures such as McDonald's omega, Cronbach's alpha, and Guttman's lambda 6. Construct validity was supported by factor analyses, with RMSEA approaching zero and CFI/TLI exceeding the 0.95 benchmark. The scale's multidimensional structure was validated, corresponding to its theoretical notion. The ORWB scale is a significant advancement in pediatric obesity, offering healthcare providers, researchers, and policymakers a comprehensive tool to evaluate and enhance the well-being of children affected by obesity.

## 1. Introduction

To create a new scale for measuring obesity-related well-being (ORWB) in children, it is crucial to incorporate data from a wide range of studies to understand the various ways in which obesity affects the lives of children. Although there have been advancements in adolescent health, the difficulties in effectively addressing obesity and its related health consequences continue to exist [1]. The research conducted by Bolling et al. [2] and Calcaterra & Zuccotti [3] emphasizes the necessity of implementing efficient approaches to address extreme obesity in children, such as metabolic and bariatric surgery. These medical methods, which are important in their own right, serve as a foundation for understanding the medical interventions in obesity management, yet they highlight the necessity for supplementary instruments that thoroughly evaluate the overall well-being of children.

The interplay between obesity and psychosocial factors is additionally impacted by societal norms and policy frameworks. In their study, Chung et al. [4] analyzed how inequalities in childhood obesity are portrayed in Australian health policy. They proposed the development of an ORWB scale that takes into account sociopolitical factors and encompasses a wider range of health determinants. Fasoulas et al. [5] established a correlation between dietary patterns and obesity, as well as oral health issues, highlighting the interdependence of lifestyle determinants on overall health. This interdependence highlights the need for a comprehensive assessment tool that evaluates not only physical health but also integrates the psychosocial aspects influenced by lifestyle choices.

Gromada et al. [6] examined the factors that impact the well-being of children in wealthy nations, including socioeconomic position and family dynamics. Herrán & Herrán-Fonseca [7] examined the correlation between meal occasions and obesity, emphasizing the impact of family and culture on dietary habits. These studies collectively suggest that obesity in children is not just a clinical issue but also a societal one, where environmental and familial factors play a crucial role. The impact of school closures during the COVID-19 pandemic on children, as examined by Hoffman & Miller [8] and Munasinghe et al. [9], has worsened the vulnerability of individuals with obesity, affecting both their mental and physical well-being. This recent global challenge further highlights the evolving nature of factors contributing to obesity, underscoring the need for a dynamic and responsive measurement tool.

Despite various tools assessing physical health outcomes in obese children (e.g., PedsQL, ORWELL 97), no validated instrument comprehensively captures physical, mental, and social well-being from the child's perspective. Kahathuduwa et al. [10] addressed how the high occurrence of overweight and obesity in communities, such as children with autism spectrum disorders, further complicates their health issues. According to Kwon et al. [11], educators' well-being indirectly affects children's obesity-related health outcomes by affecting the standard of early childhood education and care. These insights suggest that ORWB is multifactorial and influenced by various community and educational factors.

Lebrun-Harris et al. [12] presented a comprehensive analysis of the evolution of children's health throughout time, which significantly impacts the extent and direction of the ORWB scale. Meixner et al. [13] emphasize the importance of assessing the health-related quality of life in children who are overweight or obese, underscoring the need for an ORWB scale that encompasses both the physical and psychological aspects of well-being. The evolving characteristics of health behaviors throughout the COVID-19 pandemic [14–16] also emphasize the requirement for flexible evaluation instruments that can adapt to changing global health landscapes.

In light of these comprehensive insights from various studies, it becomes evident that the field of pediatric obesity requires a robust, multidimensional tool. This tool must encompass not only the direct health impacts of obesity but also its broader psychosocial and environmental influences. The studies conducted by Sserwanja et al. [17] and Stiglic & Viner [18] investigated the factors linked to childhood overweight and obesity, which could potentially provide valuable insights for the content of the ORWB scale. In this study, Viner et al. [19] critically examine the impact of school closures on mental health, highlighting the significance of this factor while evaluating a novel measurement instrument. Vogel et al. [20] highlight the importance of considering the well-being and COVID-19-related concerns of German children in current estimates of the overall risk and burden (ORB).

Based on these extensive research findings, which underscore the complexity of obesity's impact on children and the need for a comprehensive, validated, multidimensional ORWB scale, the study aimed to develop and validate an instrument that effectively measures the wide-ranging effects of obesity on children's well-being. This study aims to develop and validate the ORWB scale, a multidimensional tool that measures the physical, psychological, and social impacts of obesity on children.

## 2. Materials and Methods

### 2.1. Study Cohorts

The data collection process for this study was systematically implemented across four Tunisian governorates: Kef, Tunis, Ariana, and Gafsa. Around 20,000 students (19,945) from 16 schools were initially considered for the study. These schools were selected based on their adherence to predefined inclusion criteria, which align with the study's objectives. Ethical approval was obtained from the Ethics Committee of the Institute of Sport and Physical Education of Kef, Jendouba University, Tunisia (approval code PH-075/2023) (approval code PH-075/2023); reference no. 48, 33. This approval was based on the 1964 Declaration of Helsinki's ethical standards and subsequent amendments. Following the acquisition of the necessary ethical clearance and institutional approvals, a thorough assessment of eligibility was conducted. This assessment aimed to identify students who met the study's specific criteria.

Inclusion criteria were children aged 12–18 years with a BMI of ≥ 85th percentile for their age and sex, according to WHO standards. Exclusion criteria included the presence of known cognitive or developmental disorders that impaired comprehension. The pilot study involved 52 participants from one school.

Before the commencement of the study, written informed consent was obtained from all participants. For participants under the age of 16 years, written consent was obtained from both the participants themselves and their parents or legal guardians. All participants and their parents/guardians were fully informed about the study objectives, procedures, potential risks, and benefits before providing consent. Signed consent forms were collected from all participants, along with their parents or legal guardians where applicable, in accordance with local ethical guidelines and the standards outlined in the Declaration of Helsinki. Participants and their parents/guardians were informed of their right to withdraw from the study at any time without any negative consequences. Data collection was conducted using a combination of surveys, questionnaires, and anthropometric measurements, following a standardized protocol to ensure consistency in data gathering across all locations. Each method was selected for its ability to capture the required data accurately, providing comprehensive coverage of the study parameters. The age range of 12–18 years was chosen because adolescence is a critical period for physical and psychological development, during which obesity-related challenges become more pronounced and self-reporting abilities are sufficiently developed to ensure valid responses.

### 2.2. Instrument Development

In the development of the ORWB scale, a detailed and meticulous approach was employed, encompassing the creation of a theoretical framework, item generation, and initial testing, culminating in a pilot study. Here is an elaboration of the first four points of this development process:

#### 2.2.1. Establishing the Conceptual Framework

The foundation of the ORWB scale was built upon an extensive literature review. Key studies by Chen et al. [21] played a crucial role in identifying the core domains affected by obesity in children. These domains included physical health implications; psychological impacts, such as self-esteem and body image; social interactions; peer relationships; and lifestyle factors like dietary habits and physical activity. The literature review involved keyword searches (e.g., “childhood obesity,” “quality of life,” “well-being,” and “psychosocial impact”) across PubMed, Scopus, and Google Scholar from 2000 to 2023. Items covering physical health, emotional status, self-esteem, peer relationships, lifestyle habits, and school experience were extracted. The expert panel, consisting of 12 specialists, participated in two Delphi-style rounds to reach consensus.

Expert panels consisting of pediatricians, psychologists, nutritionists, and educators were consulted to ensure that the scale reflected a comprehensive and multidimensional view of well-being in the context of childhood obesity.

#### 2.2.2. Generation of Items

Based on the established framework, an initial pool of items was generated. These items were designed to be age-appropriate, using language that was easily understandable for children. The aim was to capture a wide spectrum of experiences and perceptions related to obesity, including emotional well-being, social experiences, physical symptoms, and daily living activities. The item pool underwent several rounds of review and revision by the expert panel to refine the wording, ensure relevance, and avoid potential biases.

A total of 32 items were generated based on the literature and expert input. Twelve experts (pediatricians, psychologists, nutritionists, and educators) reviewed and revised the items. Items were removed based on factor loadings < 0.50, low item-total correlations, redundancy, and expert feedback on content relevance. All items used a 5-point Likert scale ranging from 1 (“Strongly Disagree”) to 5 (“Strongly Agree”).

#### 2.2.3. Preliminary Testing and Refinement

The preliminary version of the ORWB scale was tested with a small group of children representative of the target demographic. This process involved not only administering the scale but also conducting interviews to understand how children interpreted the questions and whether they found them relatable.

#### 2.2.4. Pilot Study and Item Reduction

A pilot study was conducted with a larger sample to test the initial reliability and construct validity of the scale. This phase provided essential data on the correlation between the items and the overall scale. Based on the results of the pilot study, content validity was assessed. Four items were identified as not aligning well with the respective constructs they were intended to measure. These items either had low item-total correlations, did not load significantly on any factor, or were redundant with other items. Consequently, they were eliminated from the scale to enhance its focus and clarity. Through these initial four stages, the ORWB scale was carefully crafted and refined to ensure it effectively captured the complex and multidimensional aspects of well-being in children with obesity.

### 2.3. Anthropometric Measurements

Examiners performed anthropometric measurements proficiently in standard anthropometric techniques. For height measurement, the child was positioned on a flat surface, with feet together and weight distributed evenly. The head was aligned to ensure that the line of sight was perpendicular to the body, maintaining contact with the stadiometer's vertical board at the head, back, buttocks, and heels. Height was then measured to the nearest 0.1 cm using a Seca 206 portable stadiometer (Hamburg, Germany).

Body mass was measured with the child standing motionless on a Tanita BF-681 W electronic scale (Tokyo, Japan). The weight was centered, and the reading was recorded to the nearest 0.2 kg.

Sitting height (SH) measurements were conducted with the participant seated on a table in front of the stadiometer, as per Cameron's guidelines. The body mass index (BMI), calculated as the weight in kilograms divided by the square of the height in meters (kg/m^2^), was used to identify overweight and obesity in children. This method aligns with the World Health Organization (WHO/NCHS) criteria for these conditions [15].

### 2.4. Statistical Analysis

In exploring data normality for our study, skewness and kurtosis tests were instrumental. For non-Gaussian data, we identified thresholds of over 7 for asymmetry and 3 for kurtosis as indicative of poor psychometric sensitivity. To assess multivariate normality, we utilized the Mardia coefficient, which helped us pinpoint significant deviations.

Our factor exploratory analysis of the obesity well-being scale employed unweighted least squares with direct Oblimin rotation. To extract factors, we analyzed the polychoric correlation matrix. The adequacy of our sample was evaluated using the Kaiser–Meyer–Olkin (KMO) statistic, adhering to Hair et al.'s criterion that a KMO value above 0.60 is necessary to validate the factorial solution. In addition, we calculated the chi-square value of Bartlett's test of sphericity. We retained factors with eigenvalues greater than one and corroborated these findings with a scree plot. Items exhibiting factor loadings below 0.5 were excluded.

For confirmatory factor analysis (CFA), we opted for the maximum likelihood method for model parameter estimation. A range of indices was employed to assess the model's fit, including chi-square, chi-square/df, goodness of fit index (GFI), adjusted GFI (AGFI), comparative fit index (CFI), Tucker–Lewis index (TLI), root mean square error of approximation (RMSEA), and standardized root mean square residual (SRMR). Given the influence of large sample sizes on the chi-square fit statistic, we favored the chi-square/df ratio. Hu and Bentler's [22] recommendation of critical values above 0.90 for GFI and AGFI, and 0.95 or higher for CFI and TLI, guided our acceptance of the model. SRMR values below 0.08 and RMSEA values below 0.08 indicated a reasonable fit.

To evaluate the reliability of our instrument, we examined McDonald's *ω*, Cronbach's *α*, and Gutmann's *λ*6. This approach was chosen over the classical Cronbach *α* alone, which has faced criticism in the context of multidimensional scales [23]. For these indices, values above 0.90 signify exceptional internal consistency, values between 0.80 and 0.90 indicate good reliability, and values between 0.70 and 0.80 are considered acceptable.

Subscale scores were compared across different game types and genres using univariate variance analysis with age as a covariate, and effect sizes were estimated using partial eta squared. Following Cohen's guidelines, the eta-squared values below 0.01 signify trivial effects, 0.01 to 0.06 medium effects, 0.06 to 0.14 large effects, and above 0.14 very large effects. Significant differences were further analyzed using post hoc Bonferroni tests.

Convergent validity was assessed by computing the average variance extracted (AVE), with values exceeding 0.50 confirming validity. Discriminant validity was established using the Fornell–Larcker criterion, which involves comparing the square roots of AVE values against the correlation coefficients between latent constructs.

The correlations between the dimensions of the instrument, game addiction, and mental health parameters were evaluated using a Pearson correlation matrix. We classified these associations as low (< 0.35), moderate (0.36–0.67), and strong (> 0.67) [24].

## 3. Results

The exploratory sample consists of 185 individuals aged 12–18, with a mean age of 14.66 ± 2.173. The larger confirmatory sample of 487 also spans ages 12–18 but with a slightly higher mean age of 15.03 ± 1.80. The exploratory sample consists of 45% females and 55% males, with individuals distributed across primary school (37.30%), college (41.08%), and secondary school (21.62%). Within the same sample, the majority are categorized as overweight (83.78%), followed by obese type 1 (12.97%), and the least are obese type 2 (3.24%). The confirmatory sample shows a similar gender distribution, with 46.80% females and 53.20% males. In terms of academic levels, college attendees make up 59.8%, with primary and secondary school attendees at 19.5% and 20.7%, respectively. The BMI distribution in this sample is also predominantly overweight (82.75%), with obese type 1 at 12.53% and obese type 2 at 4.72%. (See Table 1).

We realize an EFA with Oblimin rotation. The provided results from Bartlett's test of Sphericity and the KMO measure indicate the appropriateness of your data for factor analysis. Bartlett's test yields a chi-square value of 2163.346 with 120 degrees of freedom, and the associated *p* value is less than 0.001, indicating a highly significant result. This suggests that the observed correlation matrix is not an identity matrix, indicating that the variables are related, which justifies the use of factor analysis. In addition, the KMO measure is 0.88, which is well above the acceptable threshold of 0.60, indicating that the sample is adequate and the dataset is suitable for structural detection. It is essential to note that the scale refers to well-being over the past 2 weeks, thereby establishing the time frame for item reflection.

Initially, Factor 1 dominates the unrotated solution, accounting for 45.50% of the variance. Factors 2 and 3 are less influential, contributing 13.50% and 8.00% to the variance, respectively, culminating in a total explained variance of 67%. Post-rotation, the variance distribution becomes more equitable among the factors, with Factor 1 explaining 23.00%, Factor 2 explaining 22.70%, and Factor 3 explaining 21.30%, maintaining the cumulative explained variance at 67%. This balance achieved by the Oblimin rotation suggests that the three factors are distinct but equally significant, potentially representing the different dimensions of well-being—physical, mental, and social—as intended in the scale's design. The unchanged cumulative variance post-rotation indicates a consistent total explanatory power, while the more balanced factor contributions post-rotation aid in interpretability, ensuring that no single factor overshadows the others in explaining the construct of well-being. The final structure comprises 16 items (Table 2): six items for physical well-being (I1–I6); five items for mental well-being (I7–I11) (I7: I am able to manage stress effectively; I8: I generally maintain a stable and positive mood; I9: I usually have a clear and focused mind; I10: I recover quickly from emotional setbacks; and I11: I feel happy and content with my life most of the time); and five items for social well-being (I12–I16).

**Figure 1:**
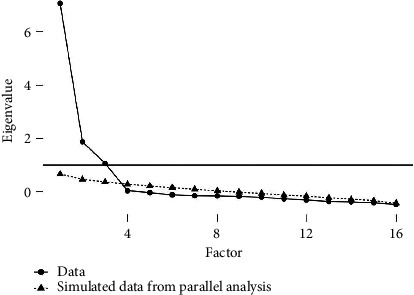
Scree plot of the ORWB.

Based on this scree plot and parallel analysis, it appears that a three-factor solution would be appropriate for your data. This aligns well with the initial intention of having three factors (physical, mental, and social well-being) (See Figure 1).

In addition, the Fruchterman–Reingold algorithm was applied to examine the items of the ORWB scale. This algorithm, by depicting factors as nodes and their correlational strengths as edges, generated a dynamic, intuitive map of the underlying factor structure of the scale. The results revealed a three-factor solution, as shown in Appendix 1 Figure 3.

### 3.1. Reliability

The Table 3 provides internal consistency coefficients for a well-being assessment instrument, encompassing physical, mental, and social well-being subscales, as well as a composite well-being index. Each subscale demonstrates high internal consistency, with McDonald's omega (*ω*) and Cronbach's alpha (*α*) exceeding 0.88 for all, and Guttman's lambda 6 (*λ*6) above 0.86, indicative of good reliability. Notably, the well-being index—which aggregates the subscales—exhibits even greater reliability (*ω* = 0.916, *α* = 0.917, *λ*6 = 0.940), suggesting an excellent internal consistency for the instrument as a whole (see Table 3)

### 3.2. CFA

**Figure 2:**
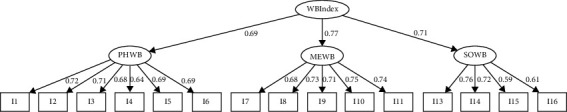
Confirmatory factor analysis of the ORWB. PHWB: Physical well-being, MEWB: Mental well-being, SOWB: Social well-being.

**Figure 3:**
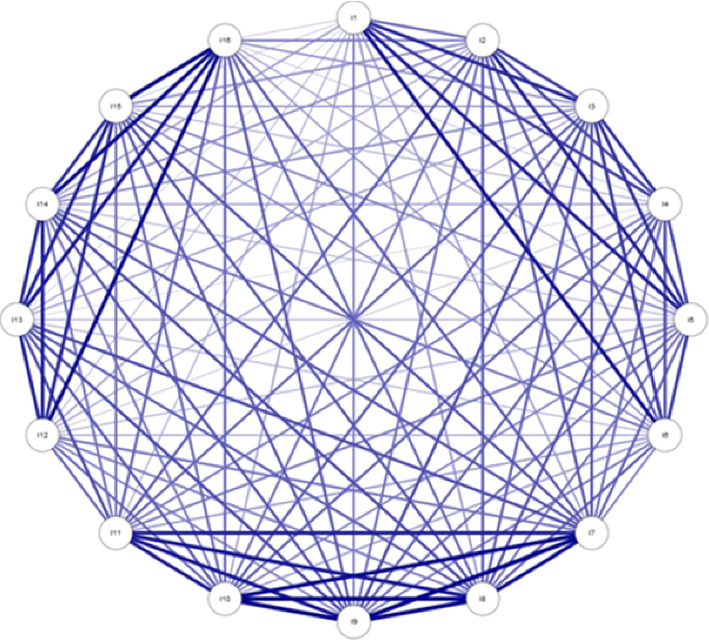
Fruchterman–Reingold algorithm.

Analyzing the fit measures for the statistical model, we see a chi-square value of 79.384 with 89 degrees of freedom and a corresponding *p* value of 0.757, indicating a good model fit as we cannot reject the null hypothesis of adequacy. Both the CFI and the TLI exceed the value of 1, typically capping at 1, which suggests an excellent fit; however, values exceeding the conventional maximum may signal overfitting or a computational anomaly. The RMSEA is 0.000, with a 90% confidence interval ranging from 0.000 to 0.018. The associated *p* value for testing a close fit is 1.000, which reinforces the model's adequacy. The SRMR value of 0.039 further supports a good fit, as it lies well below the 0.08 threshold. The model also scores high on the GFI and the AGFI, with values of 0.992 and 0.989, respectively, suggesting it accounts for the majority of variance and covariance in the data. The parsimony GFI (PGFI) is strong at 0.735, indicating a parsimonious model, and the parsimonious normed fit index (PNFI) is robust at 0.835, showing a favorable balance between model simplicity and fit (See Figure 2)

### 3.3. Discriminant and Convergent Validity

The AVE values for the constructs, based on the factor loadings, are as follows: Physical well-being (PHWB) has an AVE of 0.47, mental well-being (MEWB) has an AVE of 0.52, and social well-being (SOWB) has an AVE of 0.44. For convergent validity, AVE values should exceed 0.50, which MEWB meets, indicating strong convergent validity. However, PHWB and SOWB fall slightly short, suggesting that the items for these constructs might be refined to better capture their respective constructs. Assessing discriminant validity using the Fornell–Larcker criterion, we find that the square root of AVE for PHWB (0.69) is greater than its correlations with MEWB (0.46) and SOWB (0.35), confirming discriminant validity. Similarly, the square root of AVE for MEWB (0.72) surpasses its correlations with other factors. SOWB's square root of AVE at 0.66 is marginally greater than its correlation with MEWB (0.46), indicating adequate discriminant validity, though this is a close call and might benefit from further scrutiny. Overall, the validity measures largely affirm the model's soundness, with minor suggestions for improving the delineation of the well-being constructs (See Table 4).

## 4. Discussion

The primary aim of this study was to develop and validate the ORWB scale, a comprehensive tool for assessing well-being in obese children. The study's main results indicate that the ORWB scale demonstrates high internal consistency and construct validity, making it a reliable and effective instrument for evaluating the multifaceted impact of obesity on children's well-being.

The study's examination of the ORWB scale's internal consistency revealed impressive results, with indices such as McDonald's omega, Cronbach's alpha, and Guttman's lambda six all exceeding the 0.80 threshold. This finding is particularly significant considering the complexity of measuring multifaceted constructs like well-being in the context of pediatric obesity. The significant degree of internal consistency indicates that the ORWB scale is a reliable tool, providing consistent outcomes across diverse items intended to evaluate various facets of well-being in overweight children. In clinical practice, the reliability of a tool like the ORWB scale is paramount. Clinicians require instruments that can deliver consistent and dependable results to inform their treatment plans and monitor patient progress [25]. The high internal consistency of the ORWB scale ensures that it can be reliably used in diverse clinical scenarios, from initial assessment to tracking the outcomes of interventions over time.

The implications of these findings in academic research are similarly crucial. For researchers studying the impacts of obesity on children's well-being, the ORWB scale provides a reliable method to quantify these effects. This reliability is vital for longitudinal studies that aim to understand the long-term impact of obesity on children's physical and psychological well-being. Furthermore, the scale's reliability enhances its utility in comparative studies, where consistent measurement tools are essential for accurately comparing different populations or interventions. When compared to other scales in pediatric health [25–28], the ORWB scale is characterized by its superior reliability, as demonstrated by previous research. For example, the development of the ORWELL 97 questionnaire by Mannucci et al. [26] showed strong psychometric properties. Many scales in this field struggle to achieve such high levels of internal consistency, particularly when addressing complex, multidimensional constructs. The ORWB scale's performance in this regard sets a new benchmark for reliability in pediatric health measurement tools. This aspect of the ORWB scale's design and validation process demonstrates a rigorous approach to scale development, ensuring that the tool is theoretically sound and practically reliable.

The CFA conducted as part of this study was instrumental in establishing the construct validity of the ORWB scale. This analysis affirmed the scale's multidimensional structure, a critical aspect in accurately measuring the various facets of well-being affected by childhood obesity. The significance of a scale's multidimensionality, as noted by Varni et al. [27], lies in its ability to capture the complex and interrelated nature of health-related constructs, particularly in the context of pediatric obesity. The CFA results demonstrated strong factor loadings for each construct within the ORWB scale. These loadings indicate the extent to which each item on the scale accurately represents the underlying construct it is intended to measure. High factor loadings confirm that the items are well-aligned with their respective constructs, reinforcing the scale's overall validity. This precision in measurement is essential when dealing with multifaceted issues, such as obesity, where impacts extend not only to physical health but also to psychological and social well-being. Furthermore, the model fit indices obtained from the CFA, including the RMSEA and the CFI//TLI, were within the optimal range. These indices are crucial indicators of how well the proposed model fits the observed data. An excellent RMSEA value, close to zero, and high CFI/TLI values, exceeding the 0.95 benchmark, underscore the robustness of the ORWB scale's structure. Such strong model fit indices are indicative of a well-constructed scale that accurately captures the intended theoretical constructs. The significance of these findings is of paramount importance, particularly when comparing the ORWB scale to other instruments in the field. The ORWB scale's exceptional capacity to accurately and precisely capture the theoretical concepts of how childhood obesity affects well-being distinguishes it from other scales. The scale is validated as both a theoretically sound instrument and a practically useful tool for assessing the various implications of obesity on children's well-being. The findings align with Bronfenbrenner's ecological systems theory, which posits that interactions among individual, social, and environmental factors shape well-being. Moreover, the ORWB scale reflects key constructs from Self-Determination Theory—autonomy, competence, and relatedness—by addressing perceived control, emotional functioning, and social support.

The study's analysis of the ORWB scale's convergent validity, specifically using the AVE values, offers important insights into the scale's effectiveness. Convergent validity assesses the extent to which items of a specific construct are, in theory, related. In the case of the ORWB scale, the AVE values, especially for the mental well-being construct, were notably robust. An AVE value above the 0.50 threshold for mental well-being indicates that a significant proportion of the variance in the observed variables is accounted for by the construct. However, the AVE values for physical and social well-being constructs, though slightly below the optimal 0.50 mark, were still within acceptable limits. This suggests that while these constructs are adequately represented, there is room for further refinement to enhance the accuracy of the scale. The fact that these constructs did not reach the ideal threshold indicates a need for careful examination of the items within these constructs or potential expansion to more comprehensively capture these aspects of well-being. The findings related to convergent validity mirror the challenges highlighted in similar research, such as that conducted by Diener et al. [28]. Their work also identified instances where specific constructs within a scale did not fully achieve the desired AVE values, necessitating further refinement. These parallels underscore a common challenge in scale development, especially when dealing with complex constructs like well-being in the context of pediatric obesity. The importance of convergent validity in the ORWB scale extends beyond mere statistical significance. In practical terms, for clinicians and researchers, this means that while the scale is generally effective in measuring the intended constructs, specific attention may be required to enhance its effectiveness in comprehensively capturing all dimensions of well-being. This can lead to more precise assessments and, consequently, more targeted interventions and research inquiries.

The study's assessment of discriminant validity, using the Fornell–Larcker criterion, plays a crucial role in establishing the validity and reliability of the ORWB scale. This validity test is essential for verifying that the constructs measured by the ORWB scale are distinct and not merely reflections of one another. In the context of the ORWB scale, which aims to measure various dimensions of well-being in children with obesity, ensuring that each dimension is uniquely captured is fundamental. The successful application of the Fornell–Larcker criterion in this study indicates that each construct of the ORWB scale accounts for more variance in its indicators than in those of other constructs. This result is particularly important in multidimensional scales like the ORWB, where the risk of overlap between constructs is a common challenge. For instance, the physical and psychological aspects of well-being, although interconnected, must be measured distinctly to accurately assess the overall impact of obesity on a child's life. In their comprehensive analysis, Voorhees et al. [29] emphasized the importance of discriminant validity in scale validation. They emphasized that discriminant validity ensures the uniqueness of each construct, which is crucial for the practical application of a scale. Their study provides a detailed examination of the methods used to assess discriminant validity and provides recommendations for researchers to ensure the distinctiveness of constructs in their measurements. In the case of the ORWB scale, this validity confirms that it can effectively differentiate between the physical, psychological, and social aspects of well-being.

The testing of the ORWB scale across two distinct samples is a cornerstone of this study, highlighting the scale's robustness and generalizability. This dual-sample approach provides a comprehensive evaluation of the scale's performance, ensuring its reliability across diverse contexts. Testing a scale in multiple samples is a critical step in verifying its consistency and applicability, as emphasized by Boateng et al. [30] in their guidelines for scale development. The first sample provided an initial assessment of the scale's reliability and validity, establishing a foundation for its effectiveness. However, the true test of the scale's utility came with its application to a second, independent sample. The consistency of the ORWB scale's results across these samples is particularly noteworthy. Such consistency indicates that the scale performs reliably irrespective of sample variations, an essential attribute for a tool intended for widespread use in diverse settings. This aspect of the study is crucial for practitioners and researchers who work with different population groups. The ORWB scale's ability to yield consistent results across samples suggests that it can be effectively used in various demographic and clinical contexts, ranging from general pediatric populations to specific subgroups affected by obesity. This versatility enhances the scale's practical utility, allowing for broader applications in both clinical practice and research. Furthermore, the generalizability of the ORWB scale, as demonstrated by its performance across different samples, enhances its credibility as a reliable tool for assessing well-being in children with obesity. Generalizability is a key criterion for any assessment tool intended for widespread use, and the ORWB scale effectively meets this criterion. The comprehensive approach to testing, as mirrored in the methodology of Boateng et al. [30], not only reinforces the scale's validity but also its adaptability. It confirms that the ORWB scale can reliably capture the multifaceted nature of well-being in children with obesity across various settings and populations. The ORWB scale requires approximately 10–12 min to complete. It is suitable for administration by pediatricians, school counselors, psychologists, and researchers. Its concise format makes it feasible for school- or clinic-based screenings. The scale can also be used longitudinally to monitor changes in well-being in response to treatment or lifestyle interventions.

## 5. Limitations

While the ORWB scale has demonstrated strong psychometric properties, it is important to acknowledge certain limitations that may impact its application and interpretation. Firstly, the ORWB scale relies on self-reported measures, which inherently carry the risk of biases such as social desirability and recall bias. In children, particularly, the ability to accurately introspect and articulate feelings and experiences related to well-being can vary greatly. This limitation may affect the precision of the responses and, hence, the accuracy of the scale's findings. Future studies might consider incorporating objective measures or corroborating self-reported data with information from parents or teachers to enhance the scale's reliability. Another limitation is the cultural and socioeconomic specificity of the study's sample. While the ORWB scale was developed with a comprehensive approach, its validation in the Tunisian context may not directly translate to other cultural or socioeconomic environments. Cultural differences in the perception of obesity, lifestyle practices, and social interactions might necessitate further adaptation and validation of the scale for broader applicability. In addition, the ORWB scale's long-term applicability faces challenges because of the rapid evolution of societal norms, technology use, and lifestyle patterns, especially under the unique circumstances of the COVID-19 pandemic. The data collection for this study took place during the peak of the pandemic, a period marked by lockdowns, social distancing, and a significant shift to at-home learning. These unprecedented conditions have influenced various aspects of children's lives, potentially introducing biases in their responses related to well-being. Factors such as increased screen time, reduced physical activity, and heightened stress levels during the pandemic could have skewed the responses, reflecting the extraordinary situation rather than typical everyday experiences. As a result, some items on the ORWB scale may not fully capture the regular patterns of well-being in children or may have been disproportionately influenced by the pandemic's unique context. To ensure the scale remains relevant and effective in assessing children's well-being in the context of obesity, periodic reviews and updates will be essential. These updates should consider the potential impacts of extraordinary events, such as the COVID-19 pandemic, and adjust the scale to mitigate biases introduced by such global crises.

## 6. Conclusion

The development and validation of the ORWB scale represent a significant stride in understanding and addressing the multidimensional impact of obesity on children's well-being. This innovative scale, through its comprehensive and meticulous development process, has demonstrated robust psychometric properties. The ORWB scale's high internal consistency, validated through various reliability indices, and its solid construct validity, as shown by factor analyses and validity assessments, ensure that it is a reliable and valid tool for measuring the diverse aspects of well-being in children with obesity. The scale's ability to capture physical, psychological, social, and lifestyle dimensions offers a holistic view of how obesity affects children, filling a critical gap in the existing measurement tools. By providing nuanced insights into the various facets of well-being, the ORWB scale is not only a valuable resource for research and clinical assessment but also a crucial instrument for guiding effective interventions and policy-making. The scale's adaptability and relevance in diverse contexts make it a vital tool in the global effort to understand and mitigate the consequences of childhood obesity.

## Figures and Tables

**Table 1:** Study cohort characteristics (exploratory cohort [*n* = 185], confirmatory cohort [*n* = 487]).

	Exploratory sample		Confirmatory sample	
I1: I generally feel physically healthy.	Frequency	Percent	Frequency	Percent
I2: I have enough energy for everyday activities.	84	0.45	228	46.80
I3: I am satisfied with my level of physical fitness.	101	0.55	259	53.20
I4: I engage regularly in physical activities that improve my health.				
I5: I generally feel rested and rejuvenated after sleeping.	69	37.30%	95	19.5
I6: I maintain a diet that contributes to my physical well-being.	76	41.08%	291	59.8
I7: I am able to manage stress effectively.	40	21.62%	101	20.7
I8: I generally maintain a stable and positive mood.	Frequency	Percent	Frequency	Percent
I9: I usually have a clear and focused mind.	155	83.78%	403	82.75
I10: I recover quickly from emotional setbacks.	24	12.97%	61	12.53
I11: I feel happy and content with my life most of the time.	6	3.24%	23	4.72

Abbreviation: BMI, Body mass index.

**Table 2:** Descriptive statistics and factor loadings from the exploratory factor analysis in this table presents means, standard deviations, skewness, kurtosis, and factor loadings for the 16 items (I1–I16) of the ORWB scale based on the exploratory cohort (*n* = 185).

	Mean	Std. deviation	Skewness	Kurtosis	Factor loadings
I1: I generally feel physically healthy.	2.72	0.981	0.298	−0.149	0.92
I2: I have enough energy for everyday activities.	2.69	0.965	0.176	−0.299	0.58
I3: I am satisfied with my level of physical fitness.	2.71	0.990	0.138	−0.317	0.68
I4: I engage regularly in physical activities that improve my health.	2.72	0.981	0.159	−0.310	0.73
I5: I generally feel rested and rejuvenated after sleeping.	2.71	1.010	0.185	−0.241	0.85
I6: I maintain a diet that contributes to my physical well-being.	2.69	0.919	0.142	−0.126	0.82
I7: I am able to manage stress effectively.	2.62	0.988	0.355	0.033	0.81
I8: I generally maintain a stable and positive mood.	2.65	1.017	0.436	−0.050	0.81
I9: I usually have a clear and focused mind.	2.65	1.043	0.426	−0.129	0.83
I10: I recover quickly from emotional setbacks.	2.61	1.011	0.460	−0.040	0.93
I11: I feel happy and content with my life most of the time.	2.61	1.006	0.177	−0.394	0.76
I12: I am satisfied with my personal relationships.	2.75	0.888	0.146	−0.024	0.83
I13: I feel a strong sense of connection with the people around me.	2.75	0.953	0.453	−0.148	0.77
I14: I have people in my life who provide me with support when needed.	2.80	0.954	0.297	−0.017	0.77
I15: I am actively involved in my community.	2.61	1.006	0.306	−0.318	0.77
I16: I frequently engage in social activities.	2.67	0.952	0.284	−0.081	0.90

*Note:* Factor loadings were derived using principal axis factoring with oblimin rotation. All loadings exceed 0.50, indicating strong associations with their respective factors.

**Table 3:** Internal consistency coefficients for a well-being assessment instrument.

	McDonald's *ω*	Cronbach's *α*	Guttman's *λ*6
Physical well-being	0.881	0.881	0.865
Mental well-being	0.905	0.904	0.886
Social well-being	0.887	0.886	0.866
Well-being index	0.916	0.917	0.940

**Table 4:** Discriminant and convergent validity.

	AVE	PHWB	MEWB	SOWB
PHWB	0.47	0.69	0.46	0.35
MEWB	0.52	0.46	0.72	0.46
SOWB	0.44	0.35	0.46	0.66

*Note:* PHWB: Physical well-being, MEWB: Mental well-being, SOWB: Social well-being.

## Data Availability

The data that support the findings of this study are available from the corresponding author upon reasonable request.
